# Rescaling FSC curves

**DOI:** 10.1107/S2059798325008526

**Published:** 2025-10-08

**Authors:** Alexandre G. Urzhumtsev

**Affiliations:** a Centre for Integrative Biology, IGBMC (Institute of Genetics and of Molecular and Cellular Biology) CNRS–INSERM–University of Strasbourg 67404Illkirch France; b Département de Physique Université de Lorraine 54506Vandoeuvre-lès-Nancy France; National Centre for Biological Sciences-TIFR, India

**Keywords:** FSC curves, resolution shells, inverse cubic resolution, uniformly filled shells, half-maps, piecewise linear approximation

## Abstract

Similarity between two periodic functions is commonly assessed by comparing their Fourier coefficients within resolution shells. In particular, this approach is widely used in both crystallography and cryo-electron microscopy (cryoEM). The definition of these shells, that is the choice of resolution scale for their boundaries, can be guided by the specific goals of the analysis, by the expected features of the studied functions or simply by convention. In cryoEM, shell boundaries are traditionally defined uniformly in inverse resolution. This convention results in a vast imbalance in the number of Fourier coefficients per shell, which may bias statistical comparisons and can make function plots misleading. Constructing resolution shells with approximately equal numbers of Fourier coefficients can be achieved automatically by defining shell boundaries uniformly on the inverse cubic resolution scale. This transformation effectively zooms into the high-resolution region, which is typically the primary focus of analysis. For Fourier shell correlation (FSC) calculations between half-maps, the characteristic sigmoidal curves were observed to transform into profiles that permit piecewise linear interpolation, which may make FSC analysis more robust.

## Abstract 2

Calculating and plotting FSC curves on a uniform scale in inverse cubic resolution (Å^−3^) has several useful features.

## 1. Introduction

### 1.1. Comparison of Fourier coefficients in crystallography

Two real-valued periodic functions *f*_1_(**r**) and *f*_2_(**r**) in space can be compared either directly, point by point, or through the similarity of their Fourier coefficients, {F_1_(**s**) = *F*_1_(**s**)exp[*i*φ_1_(**s**)]} and {F_2_(**s**) = *F*_2_(**s**)exp[*i*φ_2_(**s**)]}, for a given set of three-dimensional indices, **s** ∈ S. Here, *F*_1_(**s**) and *F*_2_(**s**) are the amplitudes of these complex-valued coefficients and φ_1_(**s**) and φ_2_(**s**) are the respective phases. The functions *f*_1_(**r**) and *f*_2_(**r**) may represent the same type of data, correspond to experimental and model data, or even originate from different experimental techniques (Penczek, 2010 ▸).

In crystallography, where *f*_1_(**r**) and *f*_2_(**r**) are maps of electron-density distribution, the phases of the Fourier coefficients (called structure factors) of the experimental map are unknown. Therefore, to estimate the quality of an atomic model, the traditional measure is the *R* factor calculated using the amplitudes (Booth, 1945 ▸),
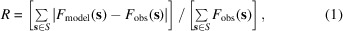
or normalized amplitudes or intensities of the Fourier coefficients (Sheldrick, 2015 ▸). In expression (1), *F*_obs_(**s**) are the experimentally obtained amplitudes of the coefficients corresponding to the electron density of the crystal, *F*_model_(**s**) are those for the map calculated from an atomic model, and phase values are ignored. When comparing two maps, therefore knowing the respective phase values, the normalized least-squares distance between two sets of complex-valued Fourier coefficients,
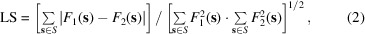
can be calculated instead (see, for example, Diamond, 1985 ▸). Being calculated over all coefficients, this function is equivalent to the least-squares distance between the maps, and inverting its sign and under the condition that the norm of the maps is fixed,
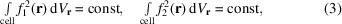
function (2) becomes equivalent to the correlation coefficient (see, for example, Lunin *et al.*, 1990 ▸),
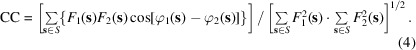
An important advantage of the coefficient (4), compared with (2), is its independence of the scale factor between the Fourier coefficients. Calculated in resolution shells, this measure (4) is known in cryoEM as the Fourier shell correlation (FSC).

In crystallography, comparison of Fourier coefficients is used not only to ‘passively’ assess map quality but also to estimate maximum-likelihood (ML) parameters for a variety of purposes, such as the calculation of ML-weighted maps (Read, 1986 ▸; Lunin & Skovoroda, 1995 ▸) and ML-based model validation and refinement (Bricogne & Irwin, 1996 ▸; Pannu & Read, 1996 ▸; Murshudov *et al.*, 1997 ▸).

### 1.2. Comparison of Fourier coefficients in cryoEM

In cryoEM, particle images are reconstructed within a large box that is virtually repeated in space. This makes it possible to analyze a discrete set of Fourier coefficients of the electrostatic scattering potential and not its continuous Fourier transform. Comparative analysis of Fourier coefficients in cryoEM, introduced as early as the 1980s (van Heel *et al.*, 1982 ▸; Saxton & Baumeister, 1982 ▸; Harauz & van Heel, 1986 ▸; van Heel, 1987 ▸), differs from crystallography in several aspects. Firstly, the coefficients are defined in a cubic box, rather than within a sphere or ellipsoid of a known resolution limit. Secondly, the phases of the coefficients are always available. Thirdly, the sets of coefficients are complete across all resolution shells, until they reach the corners of the box.

As in crystallography, correlations between these coefficients can be used to estimate how well an atomic model reproduces the experimental map. Additionally, FSC curves are widely used to evaluate the quality of image reconstruction. This is achieved by comparing the Fourier coefficients calculated from two half-maps. The latter are independent 3D reconstructions obtained from two complementary, randomly selected subsets of nearly equal size from the experimental data. A conventional measure is the lowest resolution, *d*_thresh_, for which FSC drops below a chosen threshold value. This threshold marks the point beyond which the Fourier coefficients are considered to have a low confidence. Several threshold values have been proposed and debated in the literature and online, such as 0.5 (Böttcher *et al.*, 1997 ▸; Beckmann *et al.*, 1997 ▸), 0.143 (Rosenthal & Henderson, 2003 ▸) and a criterion based on the intrinsic information content of the data (‘half-bit’; van Heel & Schatz, 2005 ▸). The discussion of these thresholds, as well as the practice of assigning *d*_thresh_ as the nominal map resolution (see Penczek, 2010 ▸; van Heel & Schatz, 2017 ▸; Sorzano *et al.*, 2017 ▸), lies beyond the scope of this article.

At the same time, Afanasyev *et al.* (2017 ▸) emphasized that ‘single-valued resolution criteria are often less relevant than the actual shape of an FSC curve’.

### 1.3. Resolution scales

The shape of a curve, in particular that for FSC, depends on the scale, which may be chosen according to the specific task. While this choice does not alter the underlying data, selecting an appropriate scale can clarify their interpretation. Indeed, different resolution scales are applied for different purposes, as implemented, in particular, in the *Phenix* suite (Liebschner *et al.*, 2019 ▸). For example, a uniform scale in Å^−2^ is optimal for calculating the Wilson plot (Wilson, 1942 ▸) to estimate the isotropic atomic displacement factor, while a uniform scale in Å^−3^ is used for computing *R* factors to validate a given model. A uniform scale in log(Å), where the number of Fourier coefficients increases proportionally between successive shells, has proven to be useful both for analyzing overall crystallo­graphic *R* factors (Urzhumtsev *et al.*, 2009 ▸) and for cryo-EM applications (Stagg *et al.*, 2014 ▸).

In cryo-EM, FSC curves are traditionally plotted as a function of the inverse resolution (Å^−1^). When calculated between half-maps, the curve typically follows a sigmoidal shape or oscillates around it. The curve begins near an FSC value of one at low resolution, gradually declines towards zero with increasing resolution, and may show an abrupt rise after crossing certain resolution limits. In this representation the number of Fourier coefficients per shell grows with the square of the inverse resolution.

Statistically, the strong imbalance in the number of Fourier coefficients per resolution shell is unsound, even though so far no major problems directly related to this have been reported. Nevertheless, visual analysis of the corresponding FSC curves may give a biased impression of the data quality. In particular, when applying cryoEM to smaller macromolecular structures, the limited number of coefficients in the lowest resolution shells may lead to misleading conclusions. Looking forward, as higher resolution reconstructions begin to reveal H atoms, visible at such resolution, these atoms will influence both high- and low-resolution data (see, for example, Afonine & Adams, 2012 ▸), analysis of which in turn requires a balanced statistical treatment across all shells. Furthermore, the anticipated development of maximum-likelihood and other statistical and validation methods in cryoEM (see, for example, Scheres, 2012 ▸; Ortiz *et al.*, 2020 ▸) will benefit from resolution shells with more homogeneous content. Even though this latter point is not directly tied to FSC curve analysis, using a consistent scaling approach seems advantageous.

Apart from historical convention, there appear to be no compelling arguments for maintaining the uniform scale in Å^−1^ traditionally used to calculate FSC curves in cryoEM. We therefore propose switching to a scale in Å^−3^, which automatically ensures a practically equal number of Fourier coefficients across resolution shells, as already used in crystallography (see, for example, Fokine & Urzhumtsev, 2002 ▸; Liebschner *et al.*, 2019 ▸). In this work, we analyze several features of this new scale.

## 2. Methods

### 2.1. Data used

As a crystallographic test case, we used experimental data for the IF2–GDP complex previously solved in our laboratory (PDB entry 4b44; Simonetti *et al.*, 2013 ▸). To ensure completeness across all resolution shells, the data were truncated at 2.9 Å resolution. Fourier coefficients (structure factors) were calculated both from the atomic model alone and with bulk-solvent correction applied (Afonine *et al.*, 2013 ▸). A control map was generated using the experimental amplitudes combined with the phases from the latter set of structure factors. To provide a closer analogy with cryo-EM calculations, the structure factors were expanded to space group *P*1.

For cryoEM analysis, we considered the HIV-1 envelope glycoprotein trimer (Ozorowski *et al.*, 2017 ▸; PDB entry 5vn3, EMDB entry EMD-8713) previously used in other methodological studies (Vilas *et al.*, 2020 ▸). Experimental maps and half-maps, along with the atomic model, were downloaded and a soft-border mask was applied around the model to filter map values outside it.

For a broader half-map analysis, several additional cryoEM data sets were taken from the EMDB (https://www.ebi.ac.uk/emdb/). For each entry, depositors had reported the resolution at which the FSC curve first reaches either 0.143 or 0.5; we denote these as *d*_0.143_ and *d*_0.5_, respectively. In addition, we retrieved alternative *d*_0.143_ and *d*_0.5_ values recalculated by the EMDB from the deposited data. These reported and recalculated values were used as references without attempting to reassess their exact significance and how they were obtained.

Entries for testing were chosen approximately at random, with the aim of covering a wide range of resolutions. Whenever possible, we selected cases where some discrepancy exists between depositor-reported and EMDB-recalculated *d*_0.143_ values. Table 1 ▸ summarizes the entries illustrated in the figures. Results for additional entries (not shown) were found to be consistent with those presented here.

### 2.2. The inverse cubic resolution scale

For a given crystal, or a virtual crystal in cryoEM, the number of Fourier coefficients up to a resolution *d*, in Å, is approximately proportional to *d*^−3^, with minor deviations due to the discrete nature of the coefficient set. Consequently, choosing resolution-shell boundaries uniformly in *d*^−3^ automatically ensures a roughly equal number of Fourier coefficients per shell. This approach also prevents an insufficient number of coefficients in the lowest resolution shells, which can occur when using the linear scale in *d*^−1^.

An additional benefit of the uniform *d*^−3^ scale is that higher resolution shells are sampled more finely compared with the conventional *d*^−1^ scale. This improves the precision in locating features of interest in FSC curves for half-maps, without increasing the total number of resolution shells. The same advantage applies when comparing maps calculated from atomic models with experimental maps.

### 2.3. Protocols used

In the tests, for each data set, we computed FSC functions using two sets of resolution shells, with boundaries defined uniformly on different resolution scales: *d*^−1^ for one set and *d*^−3^ for the other. Each FSC function was analyzed up to a resolution beyond which it showed no structural significance, either oscillating around zero or rising again after approaching zero. These upper resolution limits, *d*_high_, were chosen individually for each entry and were always higher than the respective *d*_0.143_ values reported in the EMDB.

For ease of comparison, all FSC curves presented here were computed using the same number of bins (resolution shells), *N*_shells_ = 30, although other values ranging from 10 to 100 were also tested. Varying the number of bins did not affect the observations reported below. A separate discussion is provided for the case of EMDB entry EMD-28962.

We noted the intervals where the computed FSC curves first crossed the thresholds of 0.143 and 0.5 (Table 1 ▸), although an identification of the respective resolution values was not the primary goal. No interpolation within these intervals was performed. These intervals were compared with the corresponding *d*_0.143_ and *d*_0.5_ values reported in the EMDB.

On the conventional *d*^−1^ scale, FSC curves computed between two half-maps typically display a sigmoidal shape. Nonlinearly transforming the resolution scale from *d*^−1^ to *d*^−3^ modifies the curves, potentially highlighting new features. We analyzed the transformed plots to identify any common characteristics, which, following Afanasyev *et al.* (2017 ▸), may carry structural signifiance.

## 3. Results

### 3.1. Crystallographic data for the IF2 complex

**Figure 1:**
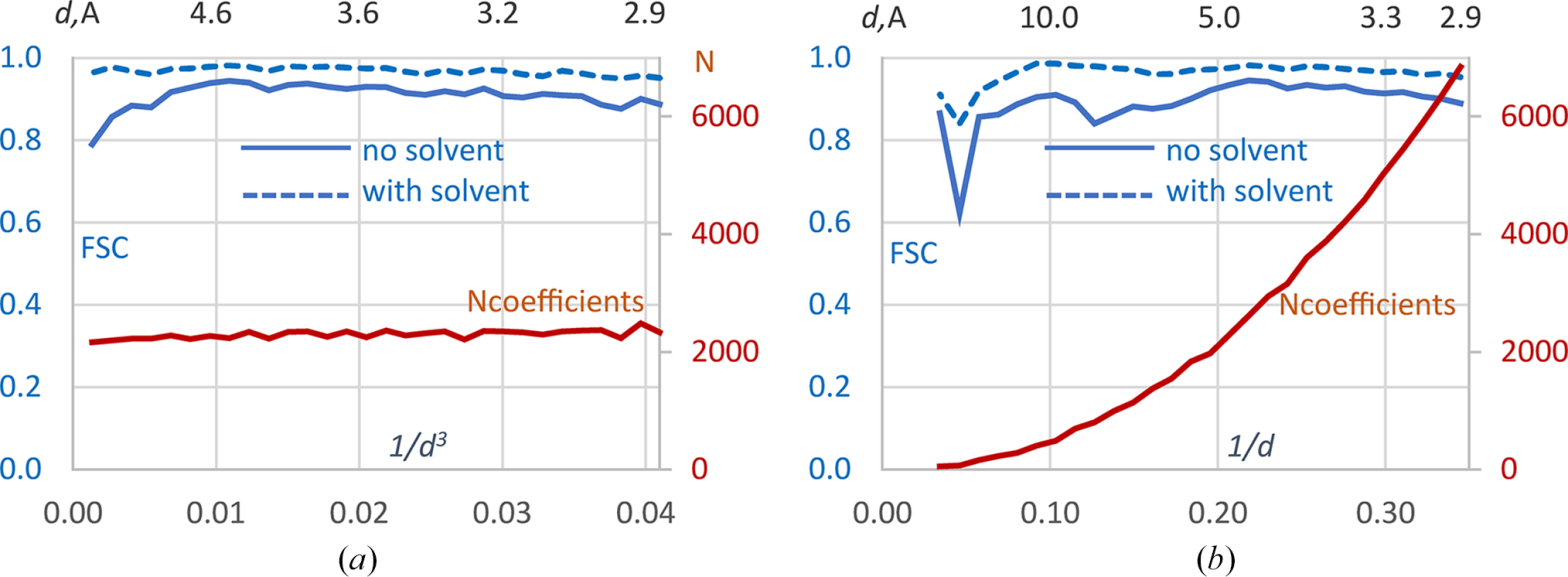
FSC curves for the IF2–GDP complex crystallographic data. Correlation values are calculated between the experimentally based map and each of two model maps, with and without bulk-solvent correction. FSC curves (in blue) were calculated in resolution shells with the boundaries chosen uniformly on the (*a*) Å^−3^ and (*b*) Å^−1^ scales. The upper axis shows the resolution recalculated in Å. Red curves show the number of the Fourier coefficients in resolution shells (scale on right).

Using crystallographic data for the IF2–GDP complex, we calculated FSC curves between the control experimentally based map and two model maps, with and without bulk-solvent correction. Fig. 1 ▸(*a*) shows these FSC curves calculated on a scale uniform in *d*^−3^ using 30 resolution shells, ensuring nearly identical numbers of Fourier coefficients per resolution shell (red curve). The FSC curves demonstrate that bulk-solvent correction is critical at resolutions lower than approximately 5 Å, although its effect is non-negligible across the entire resolution range.

Similar curves calculated on a scale uniform in *d*^−1^ with the same number of resolution shells (Fig. 1 ▸*b*) convey the same overall conclusions; however, the curve for the map without bulk-solvent correction strongly varies at low resolution. Actually, the two lowest resolution shells contain no coefficients at all and are absent from the plot, while the subsequent shells contain only a very limited number of coefficients. Without knowing this, one could incorrectly infer structural or experimental reasons for this behavior looking at the plot. Consequently, comparison of FSC values within these shells between different curves, as well as between the shells, may be ambiguous.

### 3.2. CryoEM maps and model data for the glycoprotein trimer

Next, we computed Fourier coefficients from an atomic model (PDB entry 5vn3) corresponding to the experimental cryoEM map EMDB entry EMD-8713. Coefficients were also calculated from the map itself, both as a whole and after applying a soft-border mask around the atomic model. FSC curves were then computed between the model coefficients and each of the map-derived coefficients.

**Figure 2:**
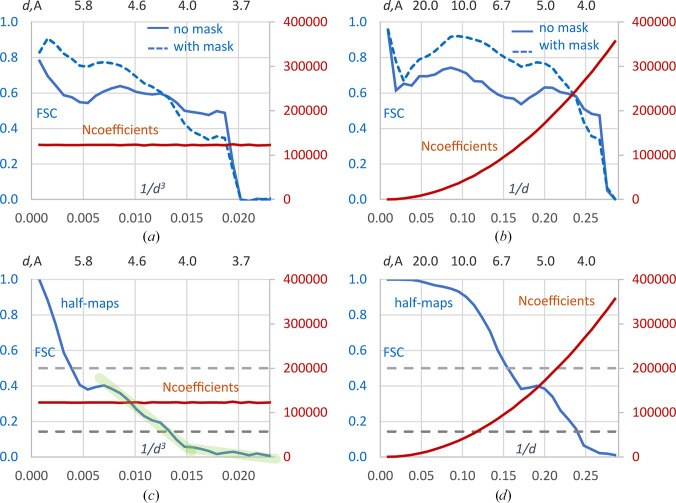
FSC curves for PDB entry 5vn3/EMDB entry EMD-8713 cryoEM data. (*a*) FSC values between the model coefficients and those calculated from the unmasked and masked experimental maps, uniform scale in Å^−3^. (*b*) The same as (*a*) but uniform scale in Å^−1^. (*c*) FSC values calculated between two experimental half-maps, uniform scale in Å^−3^. (*d*) The same as (*c*) but uniform scale in Å^−1^. Red curves show the number of the Fourier coefficients in resolution shells (scale on right). The upper axis shows the resolution recalculated in Å. Dashed lines indicate FSC values of 0.143 and 0.5. Direct lines in light green indicate two parts of the piecewise linear approximation to the FSC curve calculated on the Å^−3^ uniform scale.

On both scales and for both maps, FSC drops to zero at approximately 3.7 Å resolution (Figs. 2 ▸*a* and 2 ▸*b*). The red curve in Fig. 2 ▸(*b*), plotted on the uniform *d*^−1^ scale, illustrates the variation in the number of coefficients across the resolution shells. In this representation, FSC curves give the impression that masking significantly improves the correlation for most of the coefficients. In contrast, Fig. 2 ▸(*a*), plotted on the uniform *d*^−3^ scale with nearly equal numbers of coefficients per shell, shows that this improvement is observed for only about half of the shells and, therefore, only about half of the coefficients. It also reveals that the correlation for the lowest-resolution reflections is approximately 0.8, rather than rising to nearly 1 as Fig. 2 ▸(*b*) suggests; the apparent inflation in Fig. 2 ▸(*b*) is due to the small number of coefficients in these shells.

Finally, Fig. 2 ▸(*b*) seems to suggest that FSC drops sharply beyond 4 Å resolution, whereas Fig. 2 ▸(*a*) demonstrates that it remains around 0.5 practically up to 3.8 Å resolution, for roughly a quarter of the coefficients.

### 3.3. Half-maps for the HIV-1 glycoprotein trimer

For the same EMDB entry EMD-8713 structure, we also calculated FSC values between the experimental half-maps (Figs. 2 ▸*c* and 2 ▸*d*). This did not change the previously reported resolution values at which the FSC curve crosses the 0.5 or 0.143 thresholds. However, Fig. 2 ▸(*d*), plotted on the conventional *d*^−1^ scale, gives the misleading impression that FSC > 0.5 for about half of the data up to 3.7 Å resolution, whereas Fig. 2 ▸(*c*) on the *d*^−3^ scale shows that this actually occurs for less than 20% of these coefficients. Similarly, Fig. 2 ▸(*d*) suggests that nearly all reflections up to 3.7 Å resolution have FSC > 0.143, while Fig. 2 ▸(*c*) confirms that this is true for less than two-thirds of them.

While the formal threshold-crossing values remained the same for this example, presenting the FSC curve on the conventional *d*^−1^ scale can mislead the overall assessment of 3D reconstruction quality. In contrast, the *d*^−3^ scale representation automatically corrects for such distortions and provides a more accurate depiction of the data.

### 3.4. FSC for cryoEM half-maps

**Figure 3:**
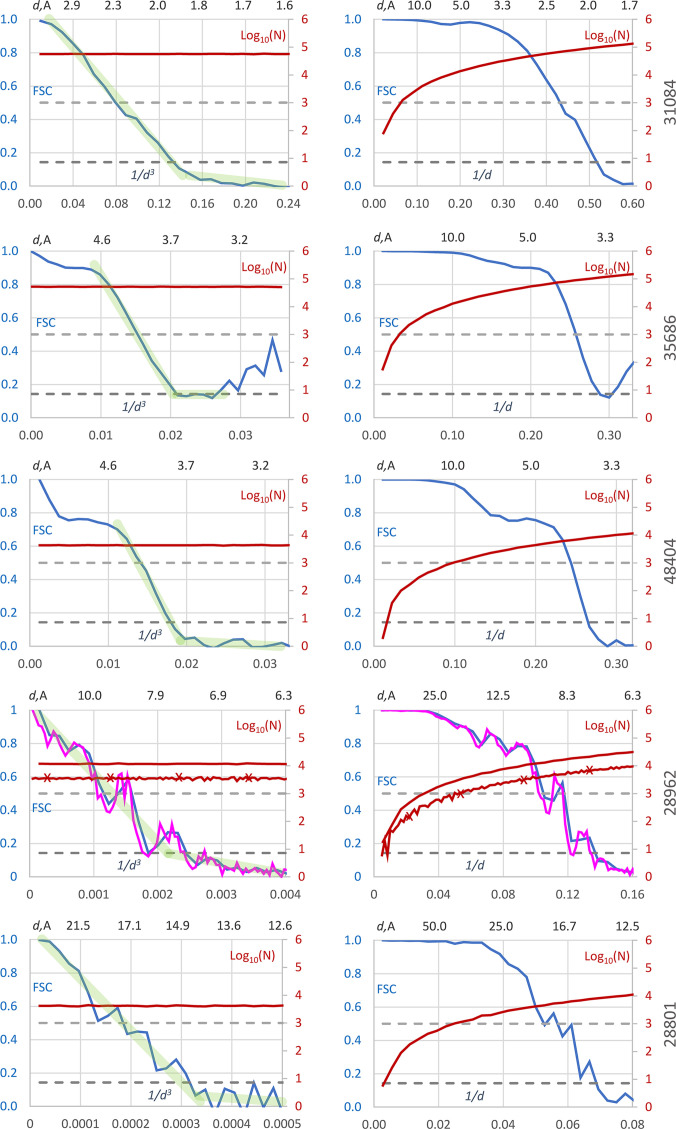
FSC curves for half-maps. FSC curves (in blue) were calculated in resolution shells with boundaries chosen uniformly on the Å^−3^ (left column) and Å^−1^ (right column) scales. The upper axis shows the resolution recalculated in Å. All curves are calculated with 30 shells, except EMDB entry EMD-28962, for which the curves in magenta additionally show the results calculated with 100 shells. Red curves represent the log_10_ of the corresponding number of Fourier coefficients (the curve with markers represents the calculation with 100 shells). Dashed lines indicate FSC values of 0.143 and 0.5. Direct lines in light green indicate two parts of the piecewise linear approximations to the FSC curves calculated on the Å^−3^ uniform scale. The EMDB code for each data set is shown on the right.

Fig. 3 ▸ shows FSC curves calculated on both scales for several other EMDB data sets: those listed in Table 1 ▸. The data sets span a range of resolutions from high to low. The plots in the right column are calculated using the conventional *d*^−1^ scale. A drastic variation in the number of Fourier coefficients is observed (their number is shown on a logarithmic scale, log_10_). Despite this variation, the number of coefficients remains large in all high-resolution shells, about 10^4^–10^5^, when using 30 shells. At the same time, it drops to only a few hundred or even tens in some low-resolution shells.

Similar to Figs. 2 ▸(*c*) and 2 ▸(*d*), the *d*^−1^-scaled plots give the misleading impression that the condition FSC > 0.143 is satisfied for nearly all Fourier coefficients across the chosen resolution range, whereas the corresponding *d*^−3^ scale plots on the left reveal that this is true for only about half of them.

Being calculated with 30 resolution shells, the resolution intervals where the FSC curves cross 0.5 or 0.143 are slightly shorter when using the *d*^−3^ scale in comparison with those using the *d*^−1^ scale (Table 1 ▸). Nevertheless, for these examples the difference is minor, indicating consistency between the scales. On both scales, these intervals generally agree with the values reported by the EMDB, although they sometimes differ from the values reported by the structure depositors. An exception is EMDB entry EMD-28962, for which the intervals where the FSC curves cross 0.143 disagree with both the deposited (6.4 Å) and EMDB-recalculated (8.27 Å) *d*_0.143_ estimates on both scales.

Specifically, for these data the FSC value in the interval 8.14–8.38 Å on the *d*^−3^ scale is 0.144 (Fig. 3 ▸*c*, row 4), while it is as large as 0.215 for the interval 8.18–8.57 Å on the *d*^−1^ scale. After switching to 100 resolution shells, calculations on both scales recovered an interval around 8.27 Å, matching the EMDB value, even though the overall curve shapes remained essentially unchanged (compare the blue and magenta curves in Fig. 3 ▸).

Examination of the plots suggests that a similar issue could be anticipated for EMDB entries EMD-35686 and EMD-28801. This is a reminder of the importance of considering rounding errors and margins when working with sharp threshold values. This may be one of the reasons for the disagreement between some values reported by the structure depositors and those recalculated by the EMDB.

This also confirms a well known fact that relying on a single value derived from a threshold of a not necessarily monotonic function, especially one calculated statistically, can lead to artifacts. Following Afanasyev *et al.* (2017 ▸), the overall shape of the FSC curve may provide more reliable and robust information.

### 3.5. Linearization of the FSC curves

Examining the FSC curve for EMDB entry EMD-8713 on the *d*^−3^ scale (Fig. 2 ▸*c*), one can observe that its decreasing part beyond the standard ‘peak and dip’ feature at about 5–6 Å resolution (see Section 3.6) can be approximated piecewise linearly. This transition between two straight lines highlights a change in FSC behavior between two regions, with the latter clearly corresponding to data of low confidence. In contrast, the curve on the *d*^−1^ scale (right plot) is typically approximated by a smooth sigmoid, with the detailed features of the curve less apparent.

A similar pattern, even more pronounced, was observed for other EMDB high- and medium-resolution data sets such as EMDB entries EMD-31084, EMD-35686 and EMD-48404 (Fig. 3 ▸). For these structures, the part of the curve beyond 5–6 Å resolution can again be described by a decreasing straight line that transitions into a horizontal, or nearly horizontal, segment, forming an L-shape.

Interestingly, the same pattern, this time for resolutions lower than 5–6 Å, can be observed for the low-resolution data sets EMDB entries EMD-28801 and EMD-28962 (Fig. 3 ▸). In all cases, interpolation smooths out the oscillations of the curve, rendering the interpolation parameters largely independent of the number of resolution shells or other eventual computing features. This property, in turn, can make the determination of critical values, such as the point of intersection of this line with a given threshold, less sensitive to computational artifacts and rounding errors, an issue that was previously encountered for EMDB entry EMD-28962.

Similar common patterns of the FSC curves for half-maps have been observed for other experimental data analyzed (not shown). At the same time, it is difficult to generalize whether this observation holds true, and to what extent, across all or even most structures. A rigorous analysis of the parameters of this FSC ‘knee’ requires careful adjustment of linear approximations to the oscillating curves and lies beyond the scope of the current study.

### 3.6. Resolution focalization

In Fig. 3 ▸, the right-hand plots for EMDB entries EMD-31804, EMD-35686 and EMD-48404 show a typical peak and dip around 5–6 Å resolution, although these features are less pronounced for the first two structures. This oscillation is generally attributed to the presence of macromolecular secondary-structure elements, such as α-helices and β-strands (see, for example, Henderson *et al.*, 2012 ▸). Such oscillations are also visible in the FSC curves calculated on the new *d*^−3^ scale (Fig. 3 ▸, left column), corresponding to the regions where the upper part of the linear approximation to the curves begins.

**Figure 4:**
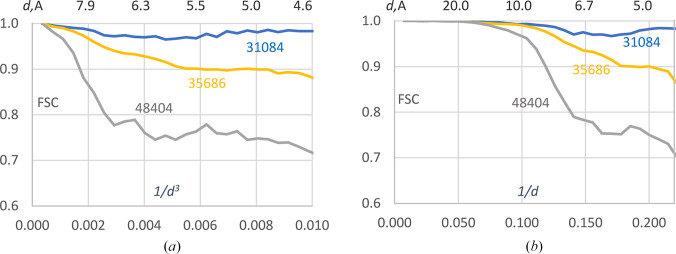
Highlighting the peak and dip of FSC curves. FSC curves calculated up to 4.5 Å resolution (0.011 Å^−3^) in resolution shells with the boundaries chosen uniformly (*a*) on the Å^−3^ scale and (*b*) on the Å^−1^ scale. The upper axis shows the resolution recalculated in Å.

Since analyzing this feature may have structural relevance, and to minimize dependence on the high-resolution data limit, the curves can be recalculated and analyzed over a standard resolution interval, for example up to 4.5 Å, which corresponds approximately to 0 ≤ *d*^−3^ ≤ 0.011 Å^−3^. Fig. 4 ▸ shows these curves for the high- and medium-resolution EMDB structures listed in Table 1 ▸.

For the same reasons as discussed above, the plots on the *d*^−3^ scale, with roughly equal numbers of Fourier coefficients per shell, appear to be more appropriate. Moreover, independently of other parameters, the resolution interval of interest, from 5 Å to approximately 8 Å, shown on this scale occupies the central part of the plot, facilitating its visualization and interpretation.

## 4. Discussion

Once a convention is established, it often becomes difficult to modify it, even when the original justification has become obsolete. One such convention in cryoEM is the calculation of FSC curves using the traditional scale in Å^−1^. A highly uneven distribution of Fourier coefficients across resolution shells caused by this choice has not prevented the identification of the resolution at which the FSC curves cross a chosen threshold.

At the same time, calculating the FSC curve on the Å^−3^ scale is computationally equivalent, yet it automatically provides a balanced distribution of Fourier coefficients per shell, yielding more statistically equivalent values than working with the conventional Å^−1^ scale. Visually inspecting the curve on this new scale allows an immediate estimate of the fraction of coefficients above a chosen FSC threshold, whereas the Å^−1^ scale interpretation can be misleading.

Tests show that this rescaling does not alter previously reported *d*_0.143_ values but produces shorter and eventually more precise intervals for future estimates of such or similar characteristics, keeping the number of resolution shells.

A more balanced distribution of Fourier coefficients allows a more reliable assessment of lower-resolution data, which is particularly important when comparing experimental maps calculated with and without masks, since masking can influence the entire resolution range but especially the medium- and low-resolution Fourier coefficients. This consideration is also relevant for future improvements of atomic models, for example by including H atoms or other ordered or partially ordered components surrounding the principal object. In contrast, analysis of FSC curves calculated using the conventional Å^−1^ scale can be strongly biased, due to the disproportionately small number of coefficients in low-resolution shells compared with higher resolution shells.

Since the FSC curves for half-maps typically exhibit a characteristic sigmoid shape, it was expected that the curves obtained after applying a common nonlinear modification would also display characteristic features. The recalculated curves preserve the typical peaks and dips at about 5–6 Å resolution when they exist. However, beyond this medium-resolution region, they decrease nearly linearly before transitioning to another region, where they oscillate around horizontal, or nearly horizontal, lines. A similar pattern has also been observed for low-resolution data sets. Using interpolated straight lines can help to avoid errors in identifying critical values such as *d*_0.143_ which may otherwise arise from an accidentally improper choice of the number of resolution shells or from small fluctuations of the FSC values within those shells. Moreover, the parameters of this piecewise linear approximation may reflect inherent properties of the data set, potentially containing more information than is actually extracted, although the significance of this remains unknown. It remains also unknown whether these randomly selected structures used in this work exhibit this feature merely by chance or whether it represents a more general trend.

The proposed modification of the resolution scale provides additional visual and numerical insights from the same data without changing the underlying calculations. While this approach requires a departure from established conventions and initially working with a less familiar curve shape, it can be viewed analogously to adopting a new software version: old options remain available, but new features offer advantages that are likely to become routine once recognized.

## Figures and Tables

**Table 1:** EMDB entries used The ‘*d*_high_’ column defines the highest resolution of the coefficients used. Intervals [0, 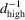] or [0, 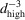] were split into resolution shells with the boundaries chosen uniformly on the respective resolution scales. The number of shells is 30 for all rows except EMD-28962*, where it is 100. The ‘depos’ and ‘emdb’ columns contain the *d*_0.143_ and *d*_0.5_ values provided by the depositors and independently recalculated by EMDB, respectively. The ‘*d*^−1^ interval’ and ’*d*^−3^ interval’ columns show the boundaries of the shells where the FSC curves first cross the values 0.143 and 0.5, respectively. The number of Fourier coefficients per shell is shown in Fig. 3 ▸.

		*d*_0.143_				*d*_0.5_			
EMDB code	*d*_high_	depos	emdb	*d*^−1^ interval	*d*^−3^ interval	depos	emdb	*d*^−1^ interval	*d*^−3^ interval
EMD-31084	1.5	1.77	1.99	1.88–1.96	1.93–1.98	1.99	2.37	2.25–2.37	2.22–2.33
EMD-35686	3.0	3.2	3.61	3.46–3.60	3.56–3.62	—	3.98	3.75–3.91	3.87–3.96
EMD-48404	3.0	3.79	—	3.75–3.91	3.78–3.86	4.10	—	4.10–4.29	4.07–4.19
EMD-28962	6.0	6.4	8.27	7.20–7.50	7.40–7.56	—	9.88	9.23–10.00	8.96–9.52
EMD-28962*	6.0	6.4	8.27	8.22–8.33	8.21–8.28	—	9.88	9.68–9.84	9.79–9.94
EMD-28801	12.0	13.8	14.73	14.4–15.0	14.5–14.8	15.75	19.80	17.1–18.0	17.3–17.9
